# Supplementary material for: Patterns of Intron Gain and Loss in Fungi
Source: PLoS Biol. 2004 Nov 30;2(12):e422. doi: 10.1371/journal.pbio.0020422 (PMC532390; doi:10.1371/journal.pbio.0020422)
Supplement: Table S1 — Also available at http://genes.mit.edu/NielsenEtAl/. (4.3 MB ZIP). [file pbio.0020422.st001.zip › NielsenEtAl/html/423.html]

AN0868.1.NCU00580.1.MG02999.1.FG08585.1


```
 CLUSTAL W (1.82) Multiple Sequence Alignments - Introns Inserted


Sequence 1: NCU00580.1	362 aa
Sequence 2: MG02999.1	359 aa
Sequence 3: FG08585.1	353 aa
Sequence 4: AN0868.1	365 aa
Alignment Length: 391 aa
Number Identitical Residues: 149 aa
Alignment Score (without introns) 7681


MG02999.1 	MGDHSNDDGAIPFPTLIRPPPEDPNKAPIENVLDVTEMTVLGR~DVFTNTRPAWQPPGAR
NCU00580.1	MGKSEEESKEAPRPTLIRPPPEDLSKAPIENTLEVIELAVIGP0NIFTNARQPWHPPGAR
FG08585.1 	MTVIEGN----VRPTLLRPPEHDEDKSLIENVLDVTELKVLGT0DIFTNTHPQWRPAGAR
AN0868.1  	MAK---------RVTLFEPPQLDPSKAQIENVLELTPVLDIGP0DVFTNTRPLWHPPGAR
          	*             **:.**  * .*: ***.*::  :  :*  ::***::  *:*.***

MG02999.1 	GIFGGSVIAMCLSAGQKTVPANFLVHSCHCYFLLAGANEVPILFHVERVRDGRSYATRTV
NCU00580.1	GIYGGAVIAMCLAAGQKTVPSDFLVHSMHCYFLLAGSAELPILFHVELVRDGRSFATRTV
FG08585.1 	GIYGGAVIAQCLAAAQKTVPETFLVHSLHCYFLLAGSSETPILYHVERVRDGRSYATRTV
AN0868.1  	GIYGGAAIAQCLSAAMRTVPADFAVHSMHCYFVLAGDSEIPILYHVERVRDGRSFITRTV
          	**:**:.** **:*. :***  * *** ****:***  * ***:*** ******: ****

MG02999.1 	QARQRGRCIFTTTVSFVKETPDAEHKENQNVVRHSASMPADAGP-----PPPDDLDEVPQ
NCU00580.1	QARQKGRCIFTTTISFVREGSSGPRGSQ---VSHASSMPIDEKTGRPCQPPPEDYDGEPE
FG08585.1 	QARQKGAAIFTTTISFVRQVSPDKKQRE---VSHAATLPENVTA------PADDWDGEPE
AN0868.1  	QARQRGRPIFTTTLSFSKANSGGKKKLE-----HASSMPS-APP------PSLPKSAESR
          	****:*  *****:** :  .   :  :     *::::*    .      *.   .  ..

MG02999.1 	LAWLG--PFQSRRIKVTNMDSP--EVHNRKSRQWTRARGKISAQGGVQAHMNALAYVSDS
NCU00580.1	LVRSGPNPFQSARIEVTNADSP--RPQDKKTRQWYRAKGKISAEGGQQAHLTALAYVSDS
FG08585.1 	WARTG--PFQSHRIEVLGASDPKVQPQDRKSRQWMRSRQKISAAGGHQAHLNALAYMSDS
AN0868.1  	FNPHGGGPFESVRDEIVNRASK---PEDKRVRRWVRCKGRISDAGAQHAHLSALAYITDS
          	    * .**:* * :: .  .     .::: *:* *.: :**  *. :**:.****::**

MG02999.1 	YFIGTISRIHKLWRFPVKVDEIDTL----------DPWVVQQIKDLH2E---------FD
NCU00580.1	YFIGTIGRIHKLWRFPFHPEDFDQL----------PEDVQERVRALH~E---------WE
FG08585.1 	YFIGTVSRLHGLWRFPFTPEEVPSL----------DEKTQEKVKEMC~E---------FE
AN0868.1  	FFIGTVSRVHEIPRFSSAAELQRALKALKNPSDLDDDDIVRALKELK~EEEAADLQRRLE
          	:****:.*:* : **.   :    *.: ...:. .     . :: :  *..::. .   :

MG02999.1 	GGG-DLKEWADRP---TLGMMVSLDHSIYFHEPRSIKADEWMFQEMESPWSGDGRGVVIQ
NCU00580.1	GLG-DPKDMVGRP---EIGMMVSLDHSIYFHEPAKVRADEWMLSEMESPWAGDGRGVVTQ
FG08585.1 	GMGSNVEDWKKRD---HVGMMVSLDHSIYFHEPTAVKADEWMFTDTESPWAGDGRGVVTQ
AN0868.1  	GALSKLEQRKKREDHKEIGMMVSLDHSIYFHNPWKFRADEWMLTEMESPWAGEGRGLVLQ
          	*  :. ::   * . . :*************:*  .:*****: : ****:*:***:* *

MG02999.1 	RIFNKEGVLVATCVQE0GIVRLKPKEEEETKAKL
NCU00580.1	RIYNKDGMLLATCVQE~GLVRLE-KNPGKTDSKL
FG08585.1 	KIFGKDGGLLATCFQE0GVVRLK-KDGGEKAAKL
AN0868.1  	KIWAKDGTLIATCTQE0GVVRLK--QDELVRAKM
          	:*: *:* *:*** ** *:***:  :     :*:
```
